# Exosomal miRNA–mRNA interactions highlight MSC-like molecular signatures in dental pulp fibroblasts

**DOI:** 10.1186/s13287-025-04884-4

**Published:** 2026-01-11

**Authors:** Koki Yoshida, Fumiya Harada, Osamu Uehara, Dedy Ariwansa, Tetsuro Morikawa, Kengo Iwasaki, Toshiyuki Nagasawa, Yoshihiro Abiko

**Affiliations:** 1 Division of Oral Medicine and Pathology, Department of Human Biology and Pathophysiology, School of Dentistry, Health Sciences University of Hokkaido, 1757 Kanazawa, Hokkaido 061-0293 Ishikari-Tobetsu, Japan; 2 Division of Oral Maxillofacial Surgery, Department of Human Biology and Pathophysiology, School of Dentistry, Health Sciences University of Hokkaido, 1757 Kanazawa, Hokkaido 061-0293 Ishikari-Tobetsu, Japan; 3 Division of Disease Control and Molecular Epidemiology, Department of Oral Growth and Development, School of Dentistry, Health Sciences, University of Hokkaido, 1757 Kanazawa, Hokkaido 061- 0293 Ishikari-Tobetsu, Japan; 4 Division of Creative and Integrated Medicine, Advanced Medicine Research Center, Translational Research Institute for Medical Innovation (TRIMI), Osaka Dental University, 8-1 Kuzuhahanazono-cho, Hirakata-shi, 573-1121 Osaka Japan; 5 Division of Periodontology and Endodontology, Department of Oral Rehabilitation, School of Dentistry, Health Sciences University of Hokkaido, 1757 Kanazawa, Hokkaido 061-0293 Ishikari-Tobetsu, Japan; 6 Division of Advanced Clinical Education, Department of Integrated Dental Education, School of Dentistry, Health Sciences University of Hokkaido, 1757 Kanazawa, Hokkaido 061-0293 Ishikari-Tobetsu, Japan

**Keywords:** Dental pulp fibroblast, Exosome, Gingival fibroblast, Mesenchymal stem cell, miRNA–mRNA interaction, Periodontal ligament fibroblast, Regenerative medicine, Transcriptome analysis

## Abstract

**Background:**

Exosomes derived from mesenchymal stem cells (MSCs) are increasingly recognized as promising mediators of tissue regeneration. However, most studies have focused on exosomes from purified MSC populations, and the regenerative relevance of exosomes secreted by fibroblast-dominant oral cell populations remains poorly understood. This study aimed to characterize the cell type–specific miRNA–mRNA regulatory features of exosomes released by gingival fibroblasts, periodontal ligament fibroblasts, and dental pulp fibroblasts, and to evaluate their potential links to MSC-like molecular programs.

**Methods:**

Fibroblast-rich cell populations were isolated from gingiva, periodontal ligament, and dental pulp tissue from the same extracted teeth, without MSC purification. Bulk RNA-seq was performed on the cells, and exosomes were collected from culture supernatants for miRNA-seq, small RNA-seq, and RNA-seq (*n* = 3 donors). Cell type–specific miRNA–mRNA regulatory axes were identified based on inverse expression patterns and confirmed using experimentally validated interactions from miRTarBase.

**Results:**

Cellular transcriptomic profiling showed that dental pulp fibroblasts expressed higher levels of genes associated with stemness, osteogenic potential, and metabolic regulation, whereas gingival and periodontal ligament fibroblasts exhibited signatures related to inflammation, vesicle trafficking, and tissue homeostasis. Exosomal RNA profiling revealed distinct regulatory modules for each fibroblast type: gingival fibroblast–derived exosomes exhibited a miR-660-5p/XKR7 axis associated with apoptosis regulation; periodontal ligament fibroblast–derived exosomes displayed a miR-199a-5p/COL19A1 axis linked to extracellular matrix remodeling; and dental pulp fibroblast–derived exosomes contained multiple MSC-associated regulatory axes, including miR-1307-3p and miR-30b-3p targeting SNRPD1, miR-493-5p targeting HMGXB4, and miR-26b-5p targeting MB–HSPD1.

**Conclusions:**

Exosomes derived from fibroblast-rich oral cell populations display distinct molecular signatures reflective of their tissue origins. Notably, exosomes from dental pulp fibroblasts exhibit MSC-like regulatory features. These findings suggest that exosomes from mixed fibroblast cultures, without requiring MSC purification, may hold promise as practical, cell-free regenerative tools, pending future functional validation.

## Background

Mesenchymal stem cells (MSCs) have shown significant potential for regenerative medicine owing to their self-renewal capacity, multipotency, and immunomodulatory functions. Among them, MSCs derived from dental pulp, periodontal ligament, and gingival tissue have garnered particular attention due to their accessibility and clinical safety [1–4]. However, the proportion of MSC-like cells, especially side population cells with stem cell-like properties, within these tissues is generally less than 1% of the total population [5], making it technically challenging to isolate and expand pure MSCs for therapeutic purposes.

To address these limitations, we adopted an approach that utilizes fibroblast-rich cell populations, namely dental pulp-derived fibroblasts (DPF), periodontal ligament-derived fibroblasts (PDLF), and gingival fibroblasts (GF), without isolating MSCs. These cell populations contain small numbers of MSC-like cells and are known to secrete a wide variety of paracrine factors, including extracellular vesicles (EVs), which contribute to tissue regeneration [6, 7].

Although these cell populations contain only a small number of MSC-like cells, fibroblasts themselves are known to secrete abundant paracrine factors and exosomes that play important roles in tissue repair and immunomodulation [8]. Moreover, exosomes secreted by MSC-like cells have been shown to possess high biological activity and to significantly contribute to tissue regeneration and repair [9]. Therefore, exosomes derived from fibroblast-rich cell populations, even without MSC purification, may contain physiologically active components that can be effectively utilized in regenerative medicine.

Exosomes, a nanosized subtype of EVs derived from the endosomal pathway, carry biomolecules such as mRNA, microRNA (miRNA), tRNA fragments, and proteins. They play crucial roles in intercellular communication and tissue homeostasis and are increasingly recognized as promising biomarkers for diagnostic and therapeutic monitoring through liquid biopsy [10, 11].

Although the regenerative applications of exosomes derived from DPF and PDLF have been studied, simultaneous and comprehensive comparisons of their exosomal RNA profiles remain extremely limited [12–14]. No study has concurrently analyzed the mRNA, miRNA, and small RNA content of exosomes derived from DPF, PDLF, and GF, all isolated from the same donor. Additionally, only one transcriptomic comparison has been reported between DPF and PDLF cells themselves, and GF has not been included in such analyses [15].

Although GF cells are known to possess MSC-like properties [4], their molecular characteristics have been far less investigated compared to DPF and PDLF. Furthermore, previous studies have shown that the RNA composition of exosomes differs from that of their parent cells, suggesting the existence of selective RNA-sorting mechanisms [16].

In this study, we conducted parallel RNA-seq, miRNA-seq, and small RNA-seq analyses on fibroblast-rich cell populations derived from DPF, PDLF, and GF, all collected and cultured from the same extracted human tooth. We also individually isolated exosomes from the culture supernatant of each cell type. This approach enabled the identification of cell-type- and exosome-specific RNA profiles, with the aim of contributing to the development of regenerative therapies for dental pulp and periodontal tissues, as well as the discovery of novel biomarkers for disease prevention.

## Methods

### Culture of human dental pulp fibroblasts, gingival fibroblasts, and periodontal ligament fibroblasts

This study was approved by the Research Ethics Committee of the School of Dentistry, Health Sciences University of Hokkaido (Approval No. 179; approved on July 12, 2019). The approved project title is “Development of extraoral periodontal tissue regeneration unit applying epigenetics to cell sheet engineering.”

Human DPFs, GFs, and PDLFs were isolated from right mandibular impacted third molars. Donor information for the three extracted teeth used in this study was as follows: a 19-year-old female, a 20-year-old female, and a 27-year-old male. All donors had no history of systemic disease, smoking, alcohol consumption, or periodontitis, and were Japanese individuals who visited the Health Sciences University of Hokkaido Dental Clinic. Written informed consent was obtained from each donor prior to sample collection.

After thorough rinsing of the extracted teeth in physiological saline, PDLF tissue was gently scraped from the middle third of the root surface using a No. 10 disposable scalpel (FEATHER Safety Razor, Osaka, Japan). GF tissue was obtained from a limited portion of marginal/attached gingival connective tissue at the cervical area, avoiding the epithelial layer. The tissue was then rinsed in physiological saline and processed for culture. Dental pulp tissue for DPF isolation was obtained by exposing the pulp chamber at the crown and extirpating the pulp using a sterilized endodontic broach (MANI, Tochigi, Japan).

All fibroblast types (DPF, GF, and PDLF) were cultured in α-MEM (NACALAI TESQUE, Kyoto, Japan) supplemented with 10% fetal bovine serum (FBS; Biowest, Nuaillé, France) and 2% Penicillin-Streptomycin-Amphotericin B Suspension (FUJIFILM Wako Pure Chemical, Osaka, Japan). Cells were seeded in 100 mm BioLite cell culture-treated dishes (Thermo Fisher Scientific, MA, USA) and incubated at 37 °C in a humidified atmosphere with 5% CO₂.

### Cellular RNA extraction and library preparation

Total RNA was extracted from cultured DPF, GF, and PDLF at passage numbers 3 using TRIzol Reagent (Thermo Fisher Scientific, Waltham, MA, USA), followed by purification with the RNeasy Mini Kit (Qiagen, Hilden, Germany) and on-column DNase digestion. RNA quality and concentration were assessed using the Agilent 2100 Bioanalyzer (Agilent Technologies, Santa Clara, CA, USA) and NanoDrop One spectrophotometer (Thermo Fisher Scientific). Only samples with OD260/280 ranging from 1.8 to 2.2, OD260/230 ≥ 1.8, and RNA Integrity Number (RIN) > 8.0 were used for sequencing.

These RNA extraction and purification steps were performed by the authors. All subsequent procedures, including strand-specific library construction using the NEBNext Poly(A) mRNA Magnetic Isolation Module and NEBNext Ultra II Directional RNA Library Prep Kit (New England Biolabs, Ipswich, MA, USA), sequencing, and read preprocessing, were outsourced to the contract analysis team at Rhelixa, Inc. (Tokyo, Japan).

### RNA sequencing and read preprocessing

RNA sequencing (RNA-seq) was performed using the Illumina NovaSeq 6000 platform (paired-end, 150 bp). Indexed libraries were quantified with a Qubit 3.0 fluorometer and assessed for size distribution using a Bioanalyzer. Libraries were pooled and sequenced on the NovaSeq platform. Base calling and demultiplexing were performed using bcl2fastq2. Raw read quality was assessed with FastQC (v0.11.9). Adapter sequences and low-quality bases were removed using Trimmomatic v0.38, and reads shorter than 36 bp after trimming were excluded.

### Read alignment and quantification

High-quality reads were aligned to the human reference genome (GRCh38/hg38) using HISAT2 v2.1.0 with default settings. Gene-level expression was quantified with featureCounts (v1.6.3), using Ensembl gene annotation (release 96) to count reads mapped to exonic regions of protein-coding genes.

### Data normalization and quality control

The resulting count matrix was analyzed using iDEP.96 [17]. Data were normalized using the Trimmed Mean of M-values (TMM) method. Genes with CPM < 0.5 in all samples were filtered out. Quality control was assessed through total read counts, scatter plots between replicates, boxplots, and density plots. These checks confirmed consistent read distributions and high comparability among samples. DESeq2 was used for differential expression analysis, and genes with adjusted *p*-values (FDR) < 0.05 were considered significant [18]. The gene-level count matrix used for this analysis was derived from the output of featureCounts and has been deposited as a CSV file (filename: Raw_Read_Count_Matrix_Fibroblasts.csv) in the Figshare repository.

### Exploratory analysis by clustering and principal component analysis

To explore sample-specific gene expression patterns, hierarchical clustering and principal component analysis (PCA) were performed using iDEP.96. For clustering, genes were first filtered by standard deviation (the 1000 most variable genes by default in iDEP.96), and Euclidean distance with complete linkage was used for genes. For samples, automatic reordering/clustering was disabled, and columns were instead arranged in the predefined order (DPF → PDLF → GF). Expression values were variance-stabilized using DESeq2, Z-score normalized per gene, and visualized in heatmaps generated by the pheatmap package in R (version 4.5.0). PCA was conducted based on the 2000 most variable genes, with the first two principal components used to visualize inter-sample relationships. To aid interpretation of group separation, 95% confidence ellipses were calculated from the covariance of PC1 and PC2 coordinates for each cell type and overlaid on the PCA plot.

### Differential gene expression analysis

Differential expression analysis between the three fibroblast groups (DPF, PDLF, GF) was conducted using DESeq2 within the cloud-based iDEP.96 platform (https://bioinformatics.sdstate.edu/idep96/). In addition to pairwise comparisons (PDLF vs. DPF, GF vs. DPF, GF vs. PDLF), a global likelihood ratio test (LRT) was applied with the design formula ~ group and reduced model ~ 1 to identify genes differentially expressed across all three groups simultaneously. Genes with adjusted *p*-values (FDR) < 0.1 and absolute log2 fold change (FC) ≥ 1 in pairwise tests, or FDR < 0.05 in the LRT, were considered differentially expressed. The number of up- and downregulated genes was visualized as bar plots. In addition, overlaps among DEGs from the three pairwise comparisons were summarized using a Venn diagram to illustrate shared and unique gene sets. The top 20 DEGs (ranked by log2FC) from each comparison were listed for interpretation and further annotation. From the LRT, 269 significant genes were obtained, and the top 100 were summarized as a heatmap to highlight global group separation.

### GO enrichment analysis of DEGs and gene clusters

Gene Ontology (GO) enrichment analysis was performed using the DEGs identified from each pairwise comparison (GF vs. PDLF, GF vs. DPF, PDLF vs. DPF) and the gene clusters obtained via k-means clustering. For DEG-based enrichment, only genes with adjusted *p*-values (FDR) < 0.1 and absolute log2 FC ≥ 1 were used. These thresholds were based on the default settings of the iDEP.96 platform. Analyses were conducted separately for upregulated and downregulated genes across GO Biological Process (BP), Cellular Component (CC), and Molecular Function (MF) categories. For pathway enrichment, a significance threshold of adjusted *p*-value < 0.01 was applied. In parallel, each k-means cluster was subjected to GO enrichment to identify functionally coherent modules.

The results summarized in Table 2 were obtained by applying GO enrichment analysis to each gene cluster (A–D), derived from k-means clustering (k = 4) of the top 2000 most variable genes by standard deviation. Functional annotation was performed using the clusterProfiler package in R (version 4.5.0), running within the Ubuntu 22.04-based iDEP.96 environment. Ensembl gene IDs were converted to Entrez IDs using the org.Hs.eg.db database. GO terms were evaluated separately for the BP, CC, and MF categories using the enrichGO() function. Adjusted *p*-values were calculated using the Benjamini–Hochberg method (FDR), with a significance threshold set at q < 0.2. Representative DEGs, including those from the top 20 pairwise comparisons as well as additional genes associated with enriched k-means pathways, were directly labeled on the figures (Fig. 5), in addition to Table 2, to facilitate interpretation.

### Exosomal RNA extraction and quality assessment

Total RNA was extracted from exosomes isolated from 10 mL of serum-free conditioned medium. This medium was collected from passage-3 DPF, GF, and PDLF cultures after 48 h in α-MEM without FBS, following a medium change from the standard culture conditions (α-MEM supplemented with 10% FBS and 2% Penicillin-Streptomycin-Amphotericin B Suspension). The medium was first centrifuged at 2,000 × g for 5 min to remove cell debris and then passed through a 0.22 μm sterile filter (Sartolab RF, Sartorius, Göttingen, Germany). These pre-clearing steps were performed by the authors, whereas all subsequent procedures were outsourced.

All subsequent procedures, including exosome capture and RNA purification with the exoRNeasy Serum/Plasma Kit (Qiagen, Hilden, Germany), together with RNA quantification, quality assessment, library preparation, sequencing, and bioinformatics analysis, were outsourced to DNA Chip Research Inc. (Kanagawa, Japan). This kit-based workflow is widely used in exosomal RNA studies [19, 20].

For quality control of extracted RNA, RNA quantity was measured using a NanoDrop One spectrophotometer (Thermo Fisher Scientific), and electrophoretic profiles were examined using an Agilent 2100 Bioanalyzer with the RNA 6000 Pico Kit.

For quality control of sequencing libraries (post-preparation), electropherograms were obtained for all nine exosomal miRNA-seq libraries using the Agilent 2100 Bioanalyzer with the High Sensitivity DNA Kit. All libraries exhibited clear peaks at approximately 160–220 bp, corresponding to adapter-ligated miRNA fragments (average ~ 190–197 bp), confirming successful small RNA library construction and consistent RNA quality across samples.

For RNA-seq libraries, Bioanalyzer traces showed appropriate fragment distributions (~ 200–1000 bp) after adapter removal and size selection, and all libraries met the provider’s quality criteria for RNA-seq, miRNA-seq, and small RNA-seq.

For small RNA-seq, library preparation was carried out using the QIAseq miRNA Library Kit (Qiagen) and QIAseq miRNA NGS 96 Index IL (Qiagen) according to the manufacturer’s instructions. This kit employs Unique Molecular Indices (UMIs) to enable digital quantification and reduce PCR bias. The average library insert sizes ranged between 190 and 197 bp as confirmed by Bioanalyzer traces.

Libraries were sequenced on the Illumina NextSeq 500/550 system using a single-end 75 bp read configuration. Each sample yielded an average of approximately 10 million reads.

Raw sequence data were subjected to adapter trimming and quality control using Trimmomatic v0.39 and FastQC v0.11.9. Summary QC statistics were compiled using MultiQC v1.14. Adapter dimers, primer-containing reads, and reads shorter than 15 bp were removed.

Trimmed reads were aligned to the human reference genome (GRCh38/hg38) using CLC Genomics Workbench v23.0.2 (Qiagen). Annotation was based on miRBase v22.1 for miRNA and GtRNAdb Release 18 for tRNA-derived fragments. Gene models from Ensembl release 96 were used for exosomal mRNA annotations.

Expression values were normalized using the TMM method, removing outlier low/high expression before sample mean centering. Clustering and PCA were performed in StrandNGS v4.0 (Agilent Technologies). Differential expression was evaluated by moderated t-tests with Benjamini–Hochberg FDR correction; significance thresholds were set at adjusted *p* < 0.05, depending on the analysis.

The processed count matrices generated from exosomal RNA-seq, miRNA-seq, and small RNA-seq analyses were used for normalization, quality control, and differential expression analyses. These datasets have been deposited in Figshare under the following filenames: Exosomal_RNA-Seq_Quantified.xlsx, Exosomal_miRNA-Seq_Quantified.xlsx, and Exosomal_smallRNA-Seq_Quantified.xlsx. The data correspond to exosomes isolated from DPF, GF, and PDLF cells.

### Processing and quality control of exosomal RNA-seq, miRNA-seq, and small RNA-seq

To assess the quality and consistency of exosomal RNA-seq, miRNA-seq, and small RNA-seq data, normalized expression values were visualized using log-transformed boxplots. For exosomal RNA-seq, transcript-level expression was normalized as Transcripts Per Million (TPM) values, which were then log-transformed using log₂(TPM + 1) and plotted per sample. For miRNA-seq and small RNA-seq, preprocessing, including UMI deduplication and normalization, was performed by the analysis vendor, DNA Chip Research Inc. The processed read counts were subsequently log-transformed [log₂(count + 1)] for visualization purposes. All samples originated from exosomes isolated from cultured DPF, GF, and PDLF cells (*n* = 3 per group). Sample labels were harmonized across the three omics layers to ensure consistency and clarity in the data presentation.

All visualizations were generated using R (version 4.5.0) and the ggplot2 package (version 3.4.0). To support transparency and reproducibility, the quantified count matrices used for these analyses have been deposited in Figshare under the following filenames: Exosomal_RNA-Seq_Quantified.xlsx, Exosomal_miRNA-Seq_Quantified.xlsx, and Exosomal_smallRNA-Seq_Quantified.xlsx. These datasets were used for normalization, quality control, and downstream differential expression analysis.

### PCA of exosomal transcriptomic profiles

PCA was performed to explore global expression patterns across exosomal mRNA-seq, miRNA-seq, and small RNA-seq datasets derived from DPF, PDLF, and GF cells (*n* = 3 per group). For each dataset, log2-transformed expression values [log2(TPM + 1) or log2(count + 1)] were used as input after excluding genes or RNAs with zero variance across samples. PCA was performed on these log2-transformed values with features standardized (scale.=TRUE) before analysis.

For exosomal mRNA-seq, TPM-normalized values were retrieved from the file Exosomal_RNA-Seq_Quantified.xlsx, and PCA was computed using the prcomp function in R (version 4.5.0). Sample labels were renamed for clarity (e.g., “D001-DPF-1” to “DPF-1”) and plotted using ggplot2 (v3.4.0).

For miRNA-seq, count data were obtained from Exosomal_miRNA-Seq_Quantified.xlsx and transformed via log2(count + 1). After filtering and normalization, PCA was conducted in the same manner. Similarly, small RNA-seq data were processed from Exosomal_smallRNA-Seq_Quantified.xlsx using log2-transformed normalized counts.

To aid interpretation of group separation, 95% confidence ellipses were calculated from the covariance of PC1 and PC2 coordinates for each cell type and overlaid on the PCA plot. In all analyses, group-specific clustering was visualized in two dimensions (PC1 vs. PC2), and samples were color-coded by cell type (DPF, PDLF, GF).

All PCA visualizations were generated in R and exported at 300 dpi for publication quality. These analyses were conducted using R version 4.5.0 with the packages ggplot2, ellipse, readxl, and dplyr. All processed datasets (Exosomal_RNA-Seq_Quantified.xlsx, Exosomal_miRNA-Seq_Quantified.xlsx, Exosomal_smallRNA-Seq_Quantified.xlsx) are available from Figshare.

### Exosomal differential expression summary and DEG barplot visualization

To summarize the results of differential gene expression analyses, we quantified the number of upregulated and downregulated genes for each pairwise comparison among the three groups (GF vs. PDLF, GF vs. DPF, and PDLF vs. DPF). Gene lists were obtained from filtered datasets meeting the following criteria: absolute FC (|Fold Change|) ≥ 2 and adjusted *p*-value (FDR) < 0.05. The data used for this analysis are available in the Figshare-deposited files: Exosomal_RNA-Seq_Quantified.xlsx, Exosomal_miRNA-Seq_Quantified.xlsx, and Exosomal_smallRNA-Seq_Quantified.xlsx.

For each omics layer (mRNA-seq, miRNA-seq, and small RNA-seq), horizontal barplots were generated using R (version 4.5.0) and the ggplot2 package (version 3.4.0). Red and blue bars indicate upregulated and downregulated genes, respectively. The number of DEGs was annotated within each bar, and the order of group comparisons was aligned with that used in the cell RNA-seq analysis for consistency.

### Exosomal DEG analysis

DEGs were identified by the contracted analysis team at DNA Chip Research Inc. based on normalized expression data derived from exosomal RNA-seq, miRNA-seq, and small RNA-seq experiments. Pairwise comparisons were conducted across the three fibroblast-derived exosome groups: DPF, GF, and PDLF. For each omics layer, upregulated and downregulated gene lists were generated for all group comparisons.

DEGs were defined using an FC threshold of ≥ 2 for upregulated genes and ≤ 0.5 for downregulated genes, along with an adjusted *p*-value < 0.05, calculated using the Benjamini–Hochberg method for false discovery rate (FDR) correction. Statistical significance was assessed using a moderated t-test with asymptotic *p*-value estimation. The full results, including statistical values, FC, and adjusted *p*-values for each gene, were provided as pre-filtered Excel files by the analysis company. Accordingly, no additional statistical filtering was performed by the authors using R.

The results of the differential expression analysis have been deposited in Figshare under the following filenames: Exosomal_RNA-Seq_data.xlsx, Exosomal_miRNA-Seq_ data.xlsx, and Exosomal_smallRNA-Seq_data.xlsx. These datasets include normalized count matrices, FC values, and statistical annotations corresponding to exosomal RNAs derived from DPF, GF, and PDLF samples.

### Integration of exosomal miRNA-seq, smallRNA-seq, and RNA-seq

To explore the relationship between miRNAs and their target mRNAs in DPF, GF, and PDLF, we conducted an integrative analysis of miRNA-seq, smallRNA-seq, and RNA-seq datasets. Differentially expressed miRNAs (DEmiRs) were first identified using edgeR with a significance threshold of FDR-adjusted *p* < 0.05 and |log2 fold change| > 1. Corresponding small non-coding RNAs (ncRNAs), such as precursor miRNAs or related entries in smallRNA-seq, were then matched by accession and Ensembl ID. To determine miRNA–mRNA relationships, we restricted targets to interactions with experimentally supported interactions curated in miRTarBase that also exhibited inverse expression trends (i.e., miRNA up, mRNA down or vice versa) [21, 22]. Prediction-only pairs were not included. The integrated results were summarized in Table 3.

### Hierarchical clustering and heatmap visualization of exosomal DEG expression

To visualize the expression patterns of DEGs among the three cell-derived exosomal RNA samples (DPF, GF, and PDLF), hierarchical clustering and heatmap analysis were performed using normalized TPM data. The DEGs were selected by applying the following criteria to the dataset Exosomal_RNA-Seq_Quantified.xlsx: adjusted *p*-value (denoted as p (Corr), a multiple testing corrected *p*-value used to control the false positive rate across multiple comparisons or tests) < 0.01 and absolute log FC > 1. This filtering was carried out based on the results summarized in Exosomal_RNA-seq_data.xlsx, and the corresponding gene list was saved as DEG_GeneIDs_Filtered_pCorr0.01_LogFC1.xlsx and deposited in Figshare.

The resulting DEG list was used to extract expression values from the TPM matrix, which were then log2-transformed and standardized into Z-scores. A hierarchical clustering heatmap was generated using the pheatmap package in R (version 4.5.0).

### Statistical analysis

The experiments were performed in triplicate for cultured cell RNA-seq and for exosomal RNA analysis. For RNA-seq of cultured cells, data were analyzed using the Wald test implemented in iDEP.96 via DESeq2 [18], and adjusted *p*-values (FDR) < 0.05 or < 0.01 were considered statistically significant, depending on the specific analysis.

For exosomal mRNA-seq data, differential expression analysis was conducted by an external service provider (DNA Chip Research Inc.) using moderated t-tests with asymptotic *p*-value estimation. Multiple testing correction was applied using the Benjamini–Hochberg false discovery rate (FDR) method, with thresholds of adjusted *p* < 0.05 or < 0.01 depending on the analysis. For miRNA-seq and small RNA-seq data, differential expression was evaluated using the edgeR package in R (version 4.5.0), applying a significance cutoff of FDR-adjusted *p* < 0.05 and absolute log2 FC > 1. All *p*-values and adjusted *p*-values reported throughout the study refer to FDR-corrected values unless otherwise stated.

## Results

### Morphology of fibroblast-rich cell populations

**Fig. 1:**
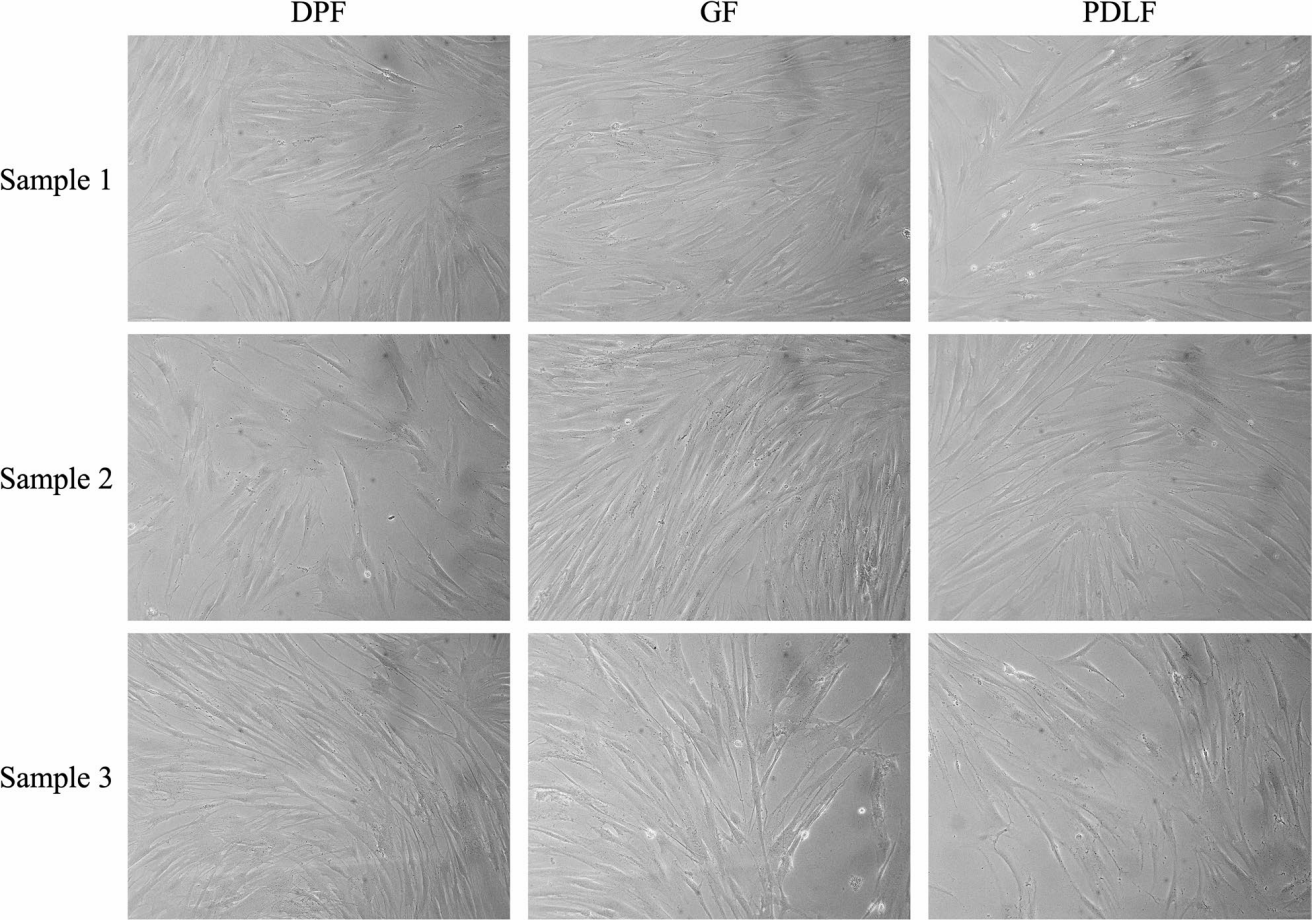
Morphology of cultured fibroblast-rich populations. Representative phase-contrast microscopy images of DPF, PDLF, and GF derived from three matched donors (Samples 1–3). All cultures exhibited typical spindle-shaped fibroblast-like morphology. Images were captured using phase-contrast microscopy (OLYMPUS, Tokyo, Japan) at ×200 total magnification (objective 10×, eyepiece 20×). For clarity, brightness and contrast were adjusted across images without altering the original data

Phase-contrast microscopy showed that the cultured cells exhibited a consistent spindle-shaped fibroblast-like morphology across all donors and tissue types (DPF, PDLF, GF) (Fig. 1). These cultures are therefore described in this study as fibroblast-rich populations that inherently include a minor fraction of MSCs.

### Data normalization ensures sample comparability in RNA-seq of cultured cells

**Fig. 2:**
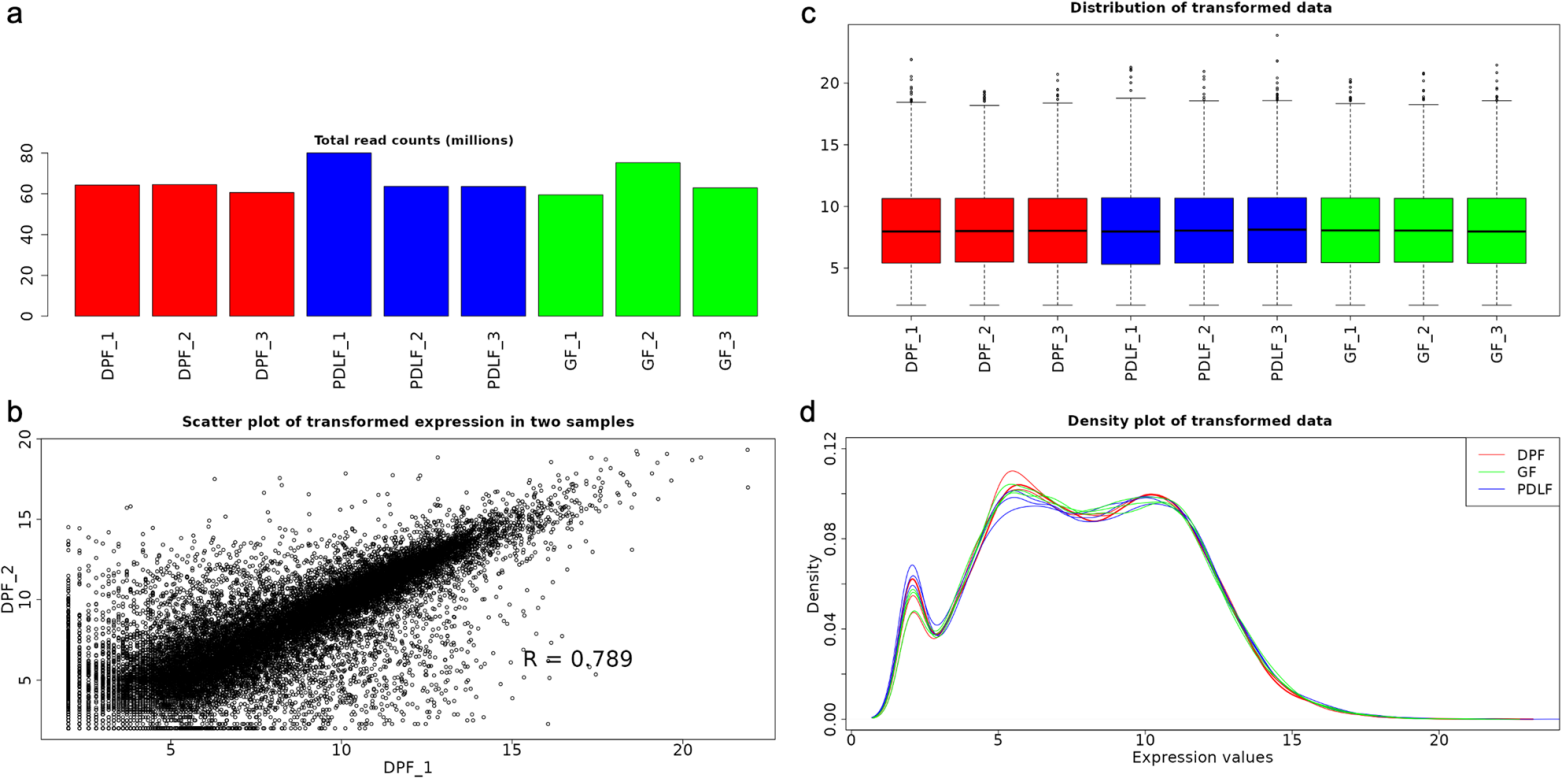
Quality control and normalization of RNA-seq data. (a) Bar plot showing total read counts (in millions) across all samples. (b) Scatter plot of transformed expression values between two biological replicates (DPF_1 vs. DPF_2). Pearson correlation coefficient (*R* = 0.789) indicates high consistency. (c) Boxplots of transformed expression values across all samples after normalization. (d) Density plots of expression values showing similar distributions among the three groups (DPF, PDLF, GF)

Total read counts per sample ranged from approximately 60 to 80 million reads, with no samples showing abnormal sequencing depth (Fig. 2a). After normalization, scatter plots revealed strong correlations between biological replicates (e.g., *R* = 0.789 as a representative correlation between DPF_1 and DPF_2), confirming reproducibility (Fig. 2b). Boxplots and density plots of transformed expression values demonstrated uniform distributions across all samples, indicating effective normalization and the absence of strong batch effects (Fig. 2c, d).

### Transcriptomic profiles reveal sample-type-specific clustering

**Fig. 3:**
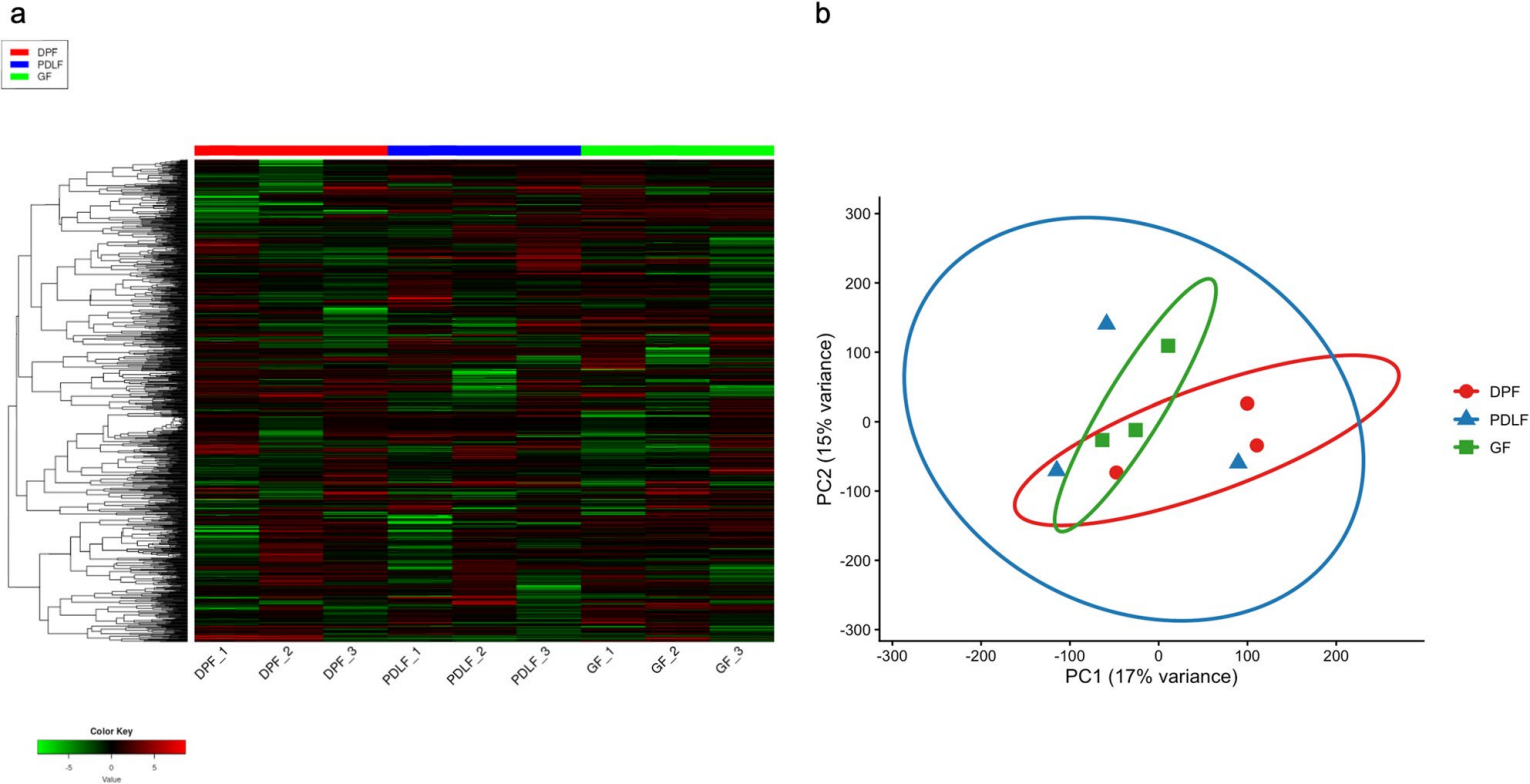
Sample clustering by hierarchical analysis and PCA. (a) Heatmap of unsupervised hierarchical clustering based on gene expression profiles from dental pulp fibroblasts (DPF, red), periodontal ligament fibroblasts (PDLF, blue), and gingival fibroblasts (GF, green). Log-transformed and Z-score normalized expression values were used. Samples grouped distinctly by cell type, forming independent clusters, indicating high intra-group similarity and clear transcriptomic separation among DPF, PDLF, and GF. (b) PCA showing sample distribution in two-dimensional space. Each sample is color-coded by cell type: DPF (red circles), PDLF (blue squares), GF (green triangles). The first and second principal components account for 17% and 15% of the total variance, respectively. The PCA indicated a tendency for separation among the three groups, with partial overlap among donors; 95% confidence ellipses are shown for each group to aid interpretation

Unsupervised hierarchical clustering of gene expression profiles indicated that samples tended to group according to cell type (DPF, PDLF, and GF), although some overlap among donors was observed (Fig. 3a). Notably, samples from the same cell type clustered together, indicating high intra-group similarity and distinct transcriptomic identities across cell types. Consistently, PCA also showed a tendency for separation among the three groups, with partial overlap among donors, along the first two principal components (PC1: 17%, PC2: 15% variance). To aid interpretation, 95% confidence ellipses were overlaid on the PCA plot (Fig. 3b).

### Transcriptomic differences among fibroblast types revealed by DEG analysis

**Fig. 4:**
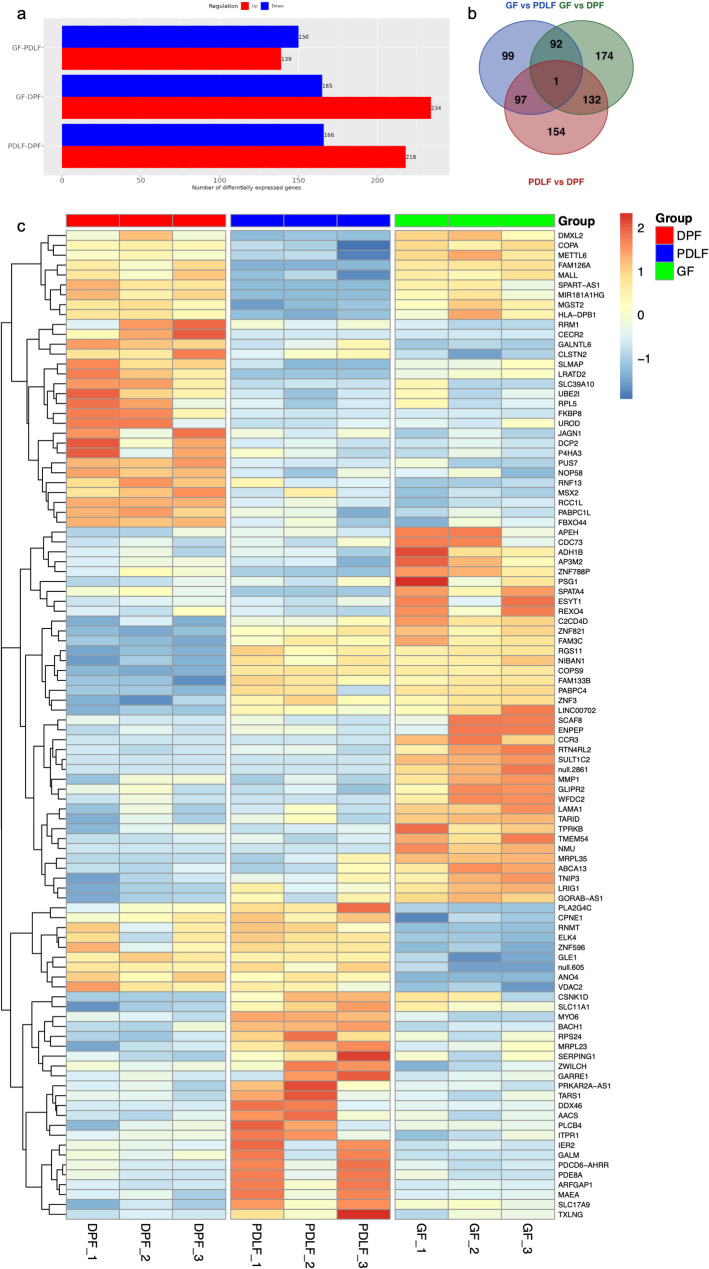
Transcriptomic Differences Among Fibroblast Types Revealed by DEG Analysis. (a) Bar plot of DEGs in pairwise comparisons. The number of upregulated (red) and downregulated (blue) DEGs is shown for each comparison among GF, PDLF, and DPF. Compared to GF, PDLF showed 139 upregulated and 150 downregulated genes. DPF showed 234 upregulated and 165 downregulated genes relative to GF. In the DPF vs. PDLF comparison, 218 genes were upregulated and 166 downregulated in DPF. (b) Venn diagram showing the overlap of DEGs among the three pairwise comparisons. Shared and unique gene sets illustrate both common and fibroblast type–specific transcriptional features. (c) Heatmaps of the top 100 DEGs across all comparisons (|log₂FC| ≥ 1, FDR < 0.1). Genes were ranked by significance and the top 100 were selected globally, rather than per comparison. Samples are shown in fixed order (DPF, PDLF, GF), and Z-score normalized expression values are displayed. Representative gene symbols are directly labeled to aid interpretation. This global heatmap highlights overall transcriptomic differences among fibroblast types, complementing the pairwise DEG counts in panel (a)

DEG analysis revealed transcriptomic differences among GF, PDLF, and DPF (Fig. 4a).

In the comparison of PDLF to GF, 139 genes were upregulated and 150 were downregulated in PDLF relative to GF. In the DPF vs. GF comparison, 234 genes were upregulated and 165 were downregulated in DPF relative to GF. The DPF vs. PDLF comparison showed 218 upregulated and 166 downregulated genes in DPF relative to PDLF. A Venn diagram was used to visualize the overlap and exclusivity of DEGs across the three pairwise comparisons (Fig. 4b), highlighting both unique and shared transcriptional signatures among fibroblast types. To further capture global transcriptional differences among the three groups simultaneously, we performed a likelihood ratio test (LRT) in DESeq2. This analysis identified 269 genes with significant differential expression (FDR < 0.05). A heatmap of the top 100 global DEGs clearly separated the three fibroblast types (Fig. 4c). Importantly, several of these top-ranked genes were also present among the pairwise DEGs (Table 1), with consistent expression trends, supporting the robustness of our dataset. The top 20 upregulated and downregulated DEGs for each pairwise comparison are listed in Table 1.

**Table 1:** Top 20 differentially expressed genes by comparison

		GF vs. PDLF			GF vs. DPF			PDLF vs. DPF		
Regulation	Rank	Symbol	log2 Fold Change	Adj.*P*val	Symbol	log2 Fold Change	Adj.*P*val	Symbol	log2 Fold Change	Adj.*P*val
Up	1	TRPM8	22.45401927	6.58E-06	LINC01436	23.49533121	1.22E-15	KCND2	22.51345095	1.83E-09
	2	TRHDE-AS1	20.87201012	4.97E-05	KCND2	22.06687569	5.37E-09	LINC01436	22.20585767	7.83E-14
	3	FAM217A	19.65514729	1.75E-04	LINC01229	21.76696273	1.18E-05	LINC01229	21.83878356	1.11E-05
	4	TMED2	11.36749689	2.02E-04	STEAP4	21.680308	1.74E-08	STEAP4	21.48808106	2.76E-08
	5	WFDC2	10.10537921	1.65E-03	TEX15	21.44699911	3.07E-09	ZIC2	21.3913787	2E-05
	6	AMD1	9.523511446	4.43E-04	ZIC2	19.05545553	2.56E-04	FIRRE	20.47501246	5.85E-05
	7	ESYT1	9.497045365	1.74E-05	FIRRE	18.01062556	7.91E-04	TEX15	20.29944908	2.76E-08
	8	IL1B	8.972729808	8.86E-03	GPC5	12.00055243	1.54E-04	CSNK1D	13.07325676	3.94E-10
	9	FAM126A	8.908663983	2.08E-12	PABPC4	11.39387613	1.01E-12	COPS9	11.05658509	3.73E-72
	10	STK19	8.646978145	8.73E-03	LSP1	11.24478059	1.13E-02	MRTFA	10.96069619	1.15E-03
	11	CLCNKB	8.621919896	4.72E-04	MRTFA	11.15159414	7.41E-04	PABPC4	10.76379937	2.96E-11
	12	HYDIN2	8.527576595	1.07E-02	COPS9	10.6987644	2.09E-67	CHODL	10.57111255	1.26E-03
	13	SULT1C2	8.521176586	5.95E-05	CSNK1D	10.56398304	2.37E-06	LINC02223	10.34573893	1.54E-03
	14	SNX27	8.377322183	4.97E-05	SLITRK4	10.53716135	2.83E-03	DDX46	10.29231778	4.71E-09
	15	FBXL15	8.227064882	1.21E-02	LINC02223	10.22321905	1.59E-03	MID1IP1	10.00042124	3.35E-06
	16	SCAF8	8.114835786	9.63E-05	ESYT1	9.7042601	6.66E-06	LINC01085	9.759185341	3.26E-04
	17	PCSK2	8.044928973	4.52E-04	TREML3P	9.697453208	4.16E-02	NRXN3	9.714293799	5.85E-03
	18	CAV2	8.015238753	2.23E-03	NUP153	9.378310109	2.14E-07	TSPAN8	9.585896432	9.54E-02
	19	P2RX4	7.945169784	8.9E-05	PAX6	9.163553421	5.68E-02	PCDH10	9.423754764	9.49E-04
	20	NHLH2	7.92470978	5.43E-02	KCNQ3	8.958892949	1.18E-02	RASSF9	9.189511193	2.54E-04
Down	1	GLB1L3	-23.29219632	2.68E-06	GLB1L3	-25.34303773	7.35E-08	TRHDE-AS1	-22.96523203	2.41E-06
	2	MIR924HG	-10.62890556	1.39E-03	UNC5C	-11.75005146	3.5E-05	FAM217A	-21.72046193	1.28E-05
	3	FCGBP	-9.918924956	1.39E-03	FKBP8	-11.53735131	1.5E-17	TRPM8	-19.84868407	1.2E-04
	4	DRAIC	-9.457203452	4.48E-03	DRAIC	-10.928097	2.86E-04	TMED2	-12.27319388	3.11E-05
	5	ANO4	-8.951532424	8.72E-07	LINC01505	-10.73195348	1.75E-03	AMD1	-12.00398294	8.8E-07
	6	DDX46	-8.723052101	1.6E-06	ANO4	-9.886636209	5.28E-09	FKBP8	-11.63041231	2.68E-17
	7	LINC01505	-8.507276679	4.12E-02	DRD1	-9.705496364	7.02E-02	CLCNKB	-9.360621703	6.79E-05
	8	PCYOX1	-8.454129773	2.68E-06	MIR924HG	-9.455433934	6.3E-03	KRT18	-9.297195921	1.55E-02
	9	PDCD6-AHRR	-8.38763216	2.12E-04	CNTNAP3	-8.853352456	2.4E-03	FBXL15	-9.233857008	2.08E-03
	10	MYO6	-8.377417109	3.55E-19	CNTNAP3P2	-8.850616023	1.14E-03	PHTF2	-8.977752165	7.54E-07
	11	BACH1	-8.264715432	2.81E-08	LINC02008	-8.817412255	4.64E-02	SPART-AS1	-8.871936266	8.8E-07
	12	TSNAX-DISC1	-8.045853363	4.35E-02	CECR2	-8.42367528	3.63E-02	LINC02008	-8.702639931	5.68E-02
	13	UNC5C	-7.997526809	2.77E-02	CRB1	-8.07191123	2.94E-02	FAM126A	-8.635891254	4.16E-12
	14	CRB1	-7.896917894	4.51E-02	CNTNAP3B	-8.054629382	6.54E-02	SNX10	-8.467824631	1.58E-02
	15	ISM2	-7.725116468	6.93E-02	FCGBP	-7.837211181	2.5E-02	CECR2	-8.308902901	4.45E-02
	16	COL9A3	-7.666340085	4.45E-02	TSNAX-DISC1	-7.79574221	4.53E-02	MARVELD1	-8.28498879	7.06E-03
	17	ELOCP2	-7.543178818	3.15E-02	PCYOX1	-7.63759483	2.89E-05	LRATD2	-8.223185655	1.11E-05
	18	MAEA	-7.527796209	4.12E-06	SPEN	-7.405164008	6.41E-06	SNX27	-8.066313637	8.97E-05
	19	ESF1	-7.270822473	5.82E-06	C8orf37-AS1	-7.261509946	8.54E-02	CAV2	-7.979494985	2.04E-03
	20	CLIC1	-7.222017589	2.73E-03	CCDC85A	-7.144728528	2.83E-03	BMP7	-7.867646857	3.03E-02

This table lists the top 20 differentially expressed genes (DEGs) identified for each pairwise comparison among the three fibroblast types: gingival fibroblasts (GF), periodontal ligament fibroblasts (PDLF), and dental pulp fibroblasts (DPF). Genes were ranked based on adjusted fold changes. The direction of regulation (up- or downregulated) is indicated relative to the first group in each comparison. “GF vs PDLF” indicates changes in GF relative to PDLF; and “GF vs DPF” indicates changes in GF relative to DPF”; PDLF vs DPF” indicates gene expression changes inPDLF relative to DPF. These DEGs highlight key genes distinguishing fibroblast subtypes

### Functional modules identified by k-means clustering

**Fig. 5:**
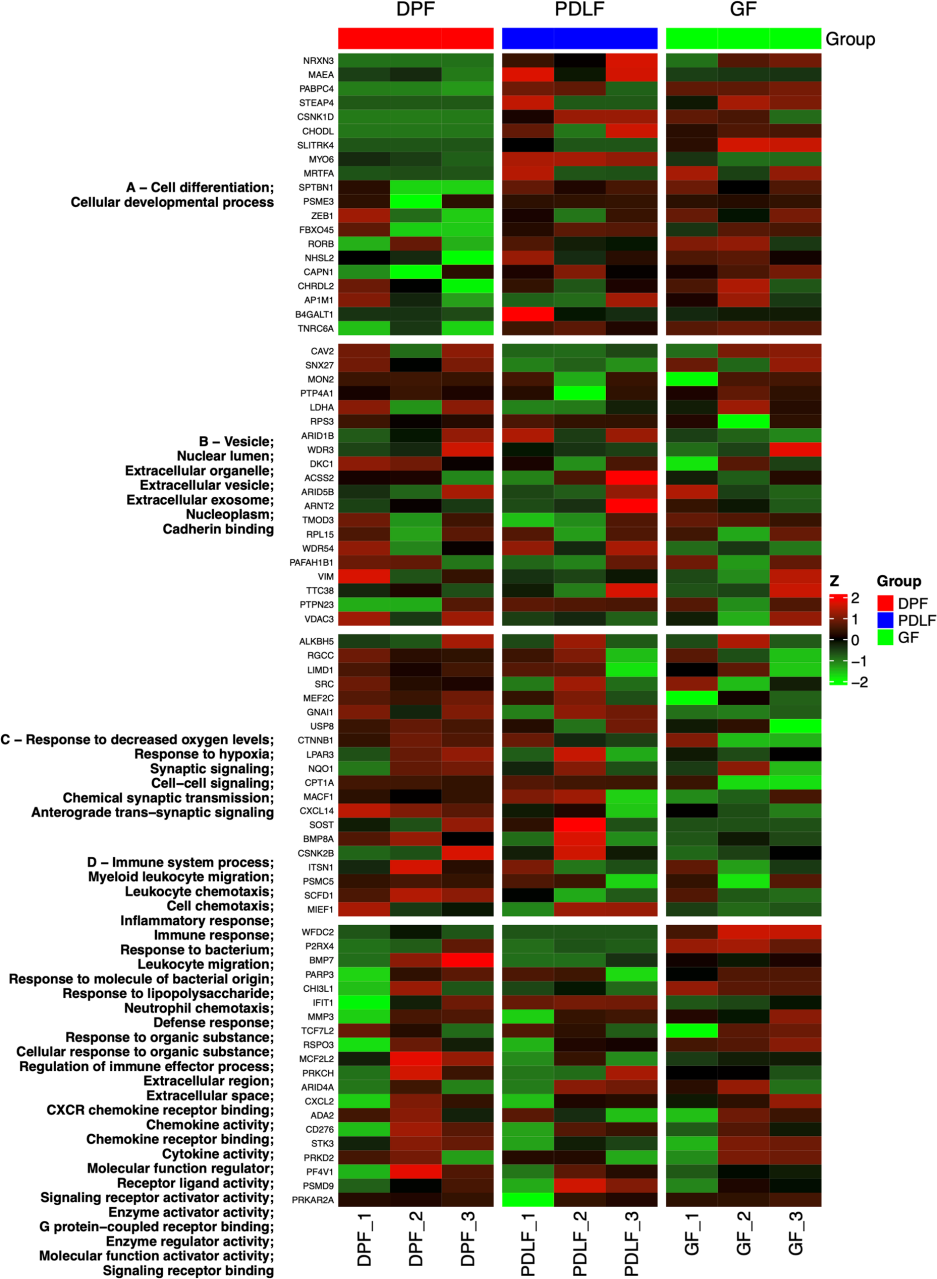
Identification of Co-expressed Gene Modules by k-means Clustering. k-means clustering of gene expression profiles identified four distinct gene clusters (Cluster A–D), each represented by a different color. The heatmap shows Z-score normalized expression levels across three fibroblast types: DPF, PDLF, and GF. Clusters represent groups of genes with similar expression patterns, and correspond to functionally enriched modules revealed by GO analysis. Representative genes were directly labeled on the heatmap. These include DEGs from the top 20 pairwise comparisons as well as additional genes associated with enriched GO pathways, thereby linking cluster-specific pathways to biologically relevant genes

To explore coordinated transcriptional programs, we applied k-means clustering to the gene expression profiles, which resulted in four distinct co-expression clusters (Fig. 5). GO enrichment analysis of each cluster revealed functionally relevant BP, CC, and MF (Table 2). Representative DEGs, including those from the top 20 pairwise comparisons as well as additional genes associated with enriched k-means pathways, were directly labeled on the figure (Fig. 5). This labeling highlights not only the most prominent DEGs but also those functionally linked to cluster-specific GO terms, thereby facilitating interpretation of biological relevance.

**Table 2:** Enriched GO terms identified in k-means clusters of genes

GO Category	Cluster	adj.Pval	nGenes	Pathways	Top 20 relevant genes in GF vs. PDLF	Top 20 relevant genes in GF vs. DPF	Top 20 relevant genes in PDLF vs. DPF
BP	A	0.004919489	151	Cell differentiation	Down in GF: MYO6, MAEA	Up in GF: STEAP4, PABPC4, MRTFA, CSNK1D, SLITRK4	Up in PDLF: STEAP4, CSNK1D, MRTFA, PABPC4, CHODL, NRXN3
	A	0.004919489	153	Cellular developmental process	Down in GF: MYO6, MAEA	Up in GF: STEAP4, PABPC4, MRTFA, CSNK1D, SLITRK4	Up in PDLF: STEAP4, CSNK1D, MRTFA, PABPC4, CHODL, NRXN3
	C	0.001305384	30	Response to decreased oxygen levels			
	C	0.00523457	27	Response to hypoxia			
	C	0.008515876	38	Synaptic signaling			
	C	0.009539922	71	Cell-cell signaling			
	C	0.009539922	36	Chemical synaptic transmission			
	C	0.009539922	36	Anterograde trans-synaptic signaling			
	D	3.36766382294362E-05	120	Immune system process	Up in GF: WFDC2, P2RX4		
	D	5.74129967089674E-05	20	Myeloid leukocyte migration	Up in GF: P2RX4		
	D	6.39943867856755E-05	20	Leukocyte chemotaxis			
	D	6.39943867856755E-05	24	Cell chemotaxis	Up in GF: P2RX4		
	D	6.48808441301468E-05	43	Inflammatory response			
	D	7.1713044169337E-05	88	Immune response	Up in GF: WFDC2		
	D	7.1713044169337E-05	34	Response to bacterium	Up in GF: WFDC2		
	D	7.1713044169337E-05	29	Leukocyte migration	Up in GF: P2RX4		
	D	0.000112905	26	Response to molecule of bacterial origin			
	D	0.000137489	25	Response to lipopolysaccharide			
	D	0.000203213	12	Neutrophil chemotaxis			
	D	0.00025167	68	Defense response	Up in GF: WFDC2		
	D	0.000252525	127	Response to organic substance	Up in GF: P2RX4		Down in PDLF: BMP7
	D	0.000301661	108	Cellular response to organic substance	Up in GF: P2RX4		Down in PDLF: BMP7
	D	0.000601495	23	Regulation of immune effector process			
CC	B	0.002622536	136	Vesicle	Up in GF: SNX27, CAV2		Down in PDLF: SNX27, CAV2
	B	0.004513463	163	Nuclear lumen	Up in GF: SNX27		Down in PDLF: SNX27
	B	0.004513463	81	Extracellular organelle			
	B	0.004513463	81	Extracellular vesicle			
	B	0.004755411	80	Extracellular exosome			
	B	0.009980906	150	Nucleoplasm	Up in GF: SNX27		Down in PDLF: SNX27
	D	0.003751488	130	Extracellular region	Up in GF: WFDC2, P2RX4		Down in PDLF: BMP7
	D	0.007709806	106	Extracellular space	Up in GF: WFDC2, P2RX4		Down in PDLF: BMP7
MF	B	0.000568301	27	Cadherin binding			
	D	2.24001577754099E-08	9	CXCR chemokine receptor binding			
	D	6.79620630195588E-07	10	Chemokine activity			
	D	1.23521183223939E-06	11	Chemokine receptor binding			
	D	8.02675129312264E-06	18	Cytokine activity			Down in PDLF: BMP7
	D	0.000317426	76	Molecular function regulator	Up in GF: WFDC2		Down in PDLF: BMP7
	D	0.000559146	23	Receptor ligand activity			Down in PDLF: BMP7
	D	0.000700113	23	Signaling receptor activator activity			Down in PDLF: BMP7
	D	0.002261972	11	Enzyme activator activity			
	D	0.003069664	17	G protein-coupled receptor binding			
	D	0.004290211	54	Enzyme regulator activity	Up in GF: WFDC2		
	D	0.00434379	12	Molecular function activator activity			
	D	0.005292284	54	Signaling receptor binding	Up in GF: P2RX4		Down in PDLF: BMP7

GO enrichment analysis was performed for each k-means gene cluster to identify overrepresented biological processes (BP), cellular components (CC), and molecular functions (MF). Adjusted *p*-values (adj.*P*val) were calculated using FDR correction. The number of genes (nGenes) contributing to each enriched term is shown. Representative DEGs from the top 20 differentially expressed genes in each fibroblast comparison (GF vs. PDLF, GF vs. DPF, and PDLF vs. DPF) are listed, with their relative expression direction (e.g., Up in GF, Down in PDLF). The direction of regulation (up- or downregulated) is indicated relative to the first group in each comparison. “GF vs. PDLF” indicates changes in GF relative to PDLF; and “GF vs. DPF” indicates changes in GF relative to DPF”; PDLF vs. DPF” indicates gene expression changes inPDLF relative to DPF

Cluster A, which was enriched for BP, showed significant enrichment in pathways related to cell differentiation and cellular developmental processes. Within these pathways, MYO6 and MAEA were found to be downregulated in GF compared to PDLF, while STEAP4, PABPC4, MRTFA, CSNK1D, and SLITRK4 were upregulated in GF compared to DPF. Additionally, STEAP4, CSNK1D, MRTFA, PABPC4, CHODL, and NRXN3 were upregulated in PDLF relative to DPF.

In Cluster D (BP category), multiple immune-related processes such as immune system process, immune response, defense response, and response to bacteria were significantly enriched. Genes including WFDC2 and P2RX4 were upregulated in GF compared to PDLF within these terms. Furthermore, P2RX4 was consistently upregulated in GF in pathways such as myeloid leukocyte migration, cell chemotaxis, and leukocyte migration. In contrast, BMP7 was downregulated in PDLF compared to DPF within terms such as response to organic substance and cellular response to organic substance.

In the CC category, Cluster B was enriched for vesicle, nuclear lumen, and nucleoplasm, where genes such as SNX27 and CAV2 were upregulated in GF and downregulated in PDLF. Cluster D showed enrichment of the extracellular region and extracellular space, with WFDC2 and P2RX4 upregulated in GF and BMP7 downregulated in PDLF.

In the MF category, Cluster D exhibited enrichment in cytokine activity, receptor ligand activity, and signaling receptor activator activity, all of which included BMP7 as a downregulated gene in PDLF. Other terms, such as MF regulator and enzyme regulator activity, included WFDC2, which was upregulated in GF and BMP7, which was downregulated in PDLF. Signaling receptor binding was also enriched in this cluster and featured P2RX4 upregulated in GF and BMP7 downregulated in PDLF.

### Identification of cell-type-specific DEGs and their association with GO clusters

Differential gene expression analysis identified a set of representative genes that were highly and specifically expressed in each fibroblast type. Below, we describe the selection criteria for these cell-type-specific genes based on directional expression patterns and their association with enriched GO terms identified via k-means clustering.

Genes were selected from the top 20 DEGs across pairwise comparisons if they showed consistent directional changes or unique, robust expression profiles (Table 1). Their functional relevance was further examined in association with enriched GO terms (Table 2).

In GF, TRPM8 and PAX6 were among the most specifically upregulated genes, together with CAV2 and SNX27. Among them, SNX27 was enriched in the GO CC terms “Vesicle,” “Nuclear lumen,” and “Nucleoplasm,” while CAV2 was enriched in “Vesicle.”

In the GF vs. PDLF comparison, the top 20 DEGs also included P2RX4, which was enriched across multiple GO categories: BP terms such as “Immune system process,” “Myeloid leukocyte migration,” “Cell chemotaxis,” “Leukocyte migration,” and “Response to organic substance”; CC terms including “Extracellular region” and “Extracellular space”; and MF terms such as “Signaling receptor binding.”

In PDLF, CLIC1, DDX46, and BACH1 were specifically elevated.

In DPF, BMP7, CECR2, and FKBP8 were distinctively upregulated. Among these, BMP7 was enriched across a wide range of GO categories, including BP terms “Response to organic substance” and “Cellular response to organic substance”; CC terms “Extracellular region” and “Extracellular space”; and MF terms such as “Cytokine activity,” “Molecular function regulator,” “Receptor ligand activity,” “Signaling receptor activator activity,” and “Signaling receptor binding.”

These representative DEGs and their functional associations are summarized in Tables 1 and 2.

### Quality control of exosomal miRNA-seq libraries

**Fig. 6:**
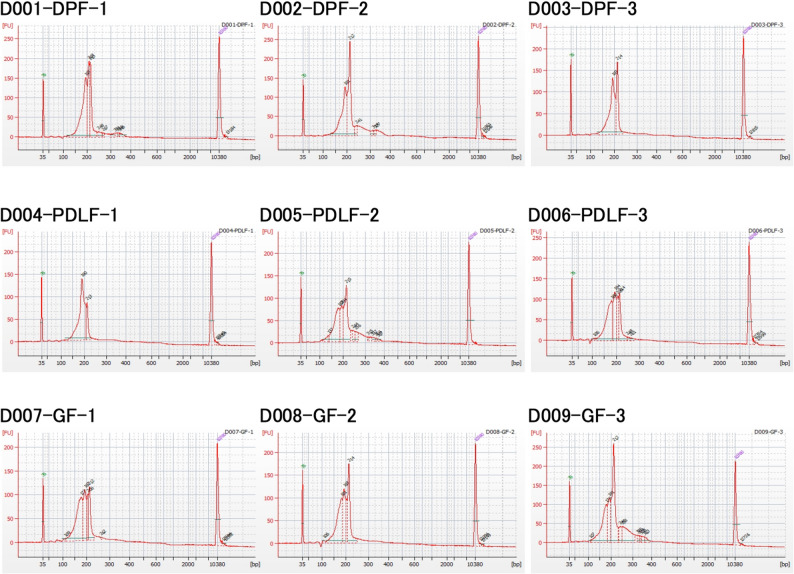
Bioanalyzer electropherograms of exosomal miRNA-seq libraries. Representative Bioanalyzer traces of exosomal miRNA-seq libraries derived from DPF, PDLF, and GF from three matched donors (*n* = 9). All libraries exhibited distinct peaks at approximately 160–220 bp, corresponding to adapter-ligated miRNA fragments, confirming consistent small RNA library quality across samples

To confirm the integrity and size distribution of the exosomal miRNA libraries prior to sequencing, electrophoretic profiles for all nine samples (DPF, PDLF, and GF; *n* = 3 per group) were obtained using the Agilent 2100 Bioanalyzer with the High Sensitivity DNA Kit (Fig. 6). All libraries exhibited a distinct peak at approximately 160–220 bp (average ~ 190–197 bp), corresponding to adapter-ligated miRNA fragments, confirming successful small RNA library construction and consistent quality across samples. These results validate the reliability of downstream sequencing and support the robustness of the comparative transcriptomic analyses.

### Global transcriptomic profiles of Exosomal RNA, miRNA, and small RNA

**Fig. 7:**
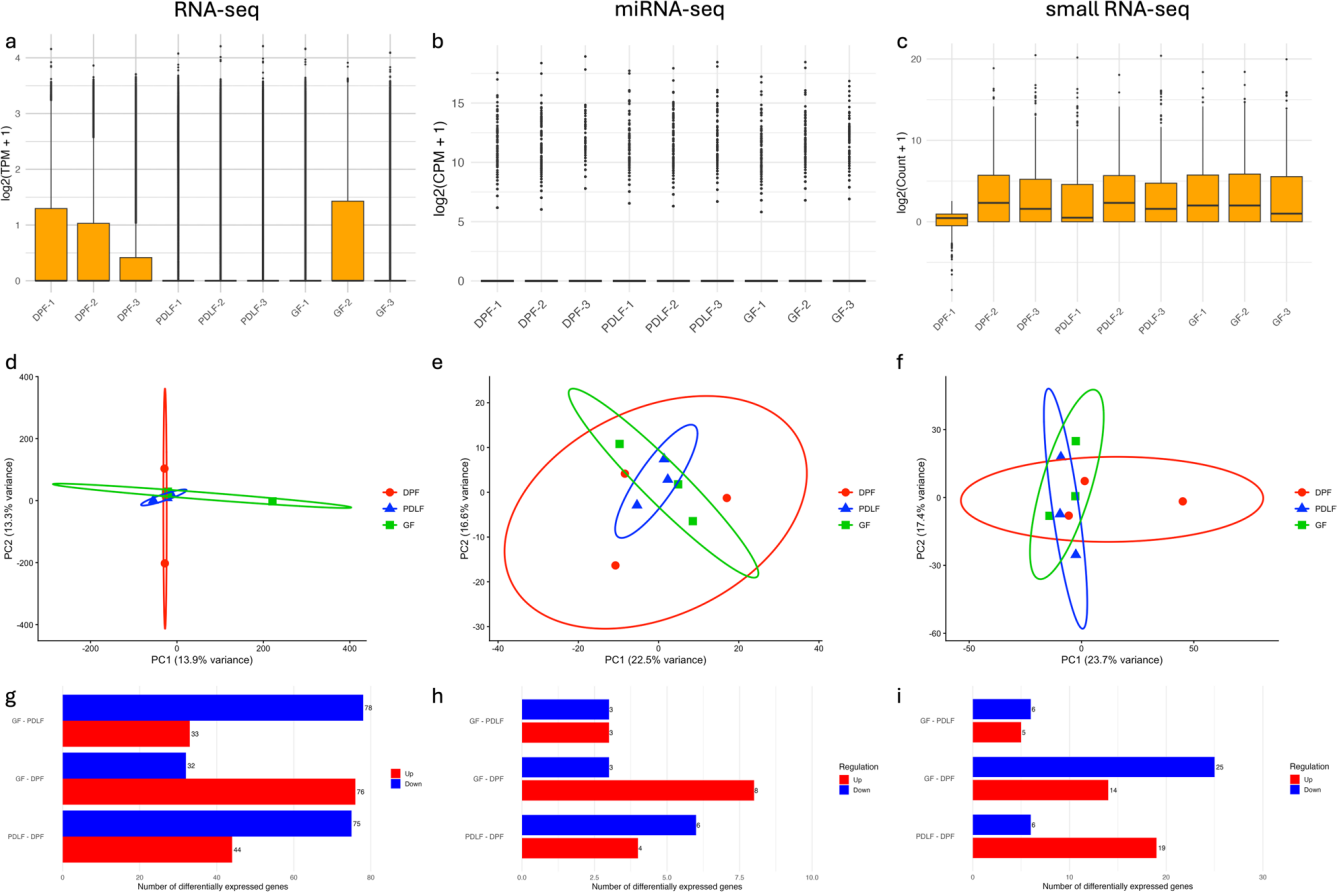
Transcriptomic profiles of GF, PDLF, and DPF in exosomal RNA-seq, miRNA-seq, and small RNA-seq. Figure 7 illustrates transcriptomic differences among GF, PDLF, and DPF based on exosomal RNA-seq (a, d, g), miRNA-seq (b, e, h), and small RNA-seq (c, f, i). Boxplots (a–c) display normalized expression distributions, confirming proper normalization and comparability among groups. PCA plots (d–f) show group-dependent clustering with partial overlap, with PC1 and PC2 explaining 13.9% and 13.3% of the variance in RNA-seq (d), 22.5% and 16.6% in miRNA-seq (e), and 23.7% and 17.4% in small RNA-seq (f), respectively. Ellipses represent the 95% confidence intervals, supporting the presence of group-specific tendencies in exosomal transcriptomic profiles. Bar plots (g–i) summarize the number of differentially expressed transcripts. In RNA-seq (g): GF vs. PDLF had 33 upregulated and 78 downregulated genes; GF vs. DPF, 76 up and 32 down; PDLF vs. DPF, 44 up and 75 down. In miRNA-seq (h): 3/3, 8/3, and 4/6, respectively. In small RNA-seq (i): 5/6, 14/25, and 19/6 small RNAs were identified

To comprehensively characterize the transcriptomic landscape among GF, PDLF, and DPF groups, we analyzed exosomal RNA using three distinct sequencing datasets: RNA-seq, miRNA-seq, and small RNA-seq (Fig. 7). Figure 7a, d, and g correspond to RNA-seq results; Fig. 7b, e, and h represent miRNA-seq; and Fig. 7c, f, and i show the findings from small RNA-seq.

The boxplots in Fig. 7a–c illustrate the distribution of expression values in each group, confirming that normalization was appropriately conducted and that intergroup comparisons within each dataset are valid.

PCA plots for RNA-seq (Fig. 7d: PC1 = 13.9%, PC2 = 13.3%), miRNA-seq (Fig. 7e: PC1 = 22.5%, PC2 = 16.6%), and small RNA-seq (Fig. 7f: PC1 = 23.7%, PC2 = 17.4%) demonstrated group-dependent clustering of GF, PDLF, and DPF samples along the first and second principal components. Although partial overlap was observed, the ellipses of the 95% confidence intervals indicated distinguishable tendencies among the three groups. These results indicate the presence of group-specific transcriptomic differences and validate the quality and appropriateness of the sequencing and analytical pipelines used.

Figure 7g–i summarize the number of differentially expressed transcripts (DETs) identified between groups. In RNA-seq (Fig. 7g), 33 upregulated and 78 downregulated genes were found in GF compared to PDLF; 76 upregulated and 32 downregulated in GF compared to DPF; and 44 upregulated and 75 downregulated in PDLF versus DPF.

In miRNA-seq (Fig. 7h), 3 upregulated and 3 downregulated miRNAs were detected in GF versus PDLF; 8 upregulated and 3 downregulated in GF versus DPF; and 4 upregulated and 6 downregulated in PDLF versus DPF.

For small RNA-seq (Fig. 7i), 5 upregulated and 6 downregulated small RNAs were observed in GF versus PDLF; 14 upregulated and 25 downregulated in GF versus DPF; and 19 upregulated and 6 downregulated in PDLF versus DPF.

### Heatmap highlights transcriptomic signatures across fibroblast-derived exosomes

**Fig. 8:**
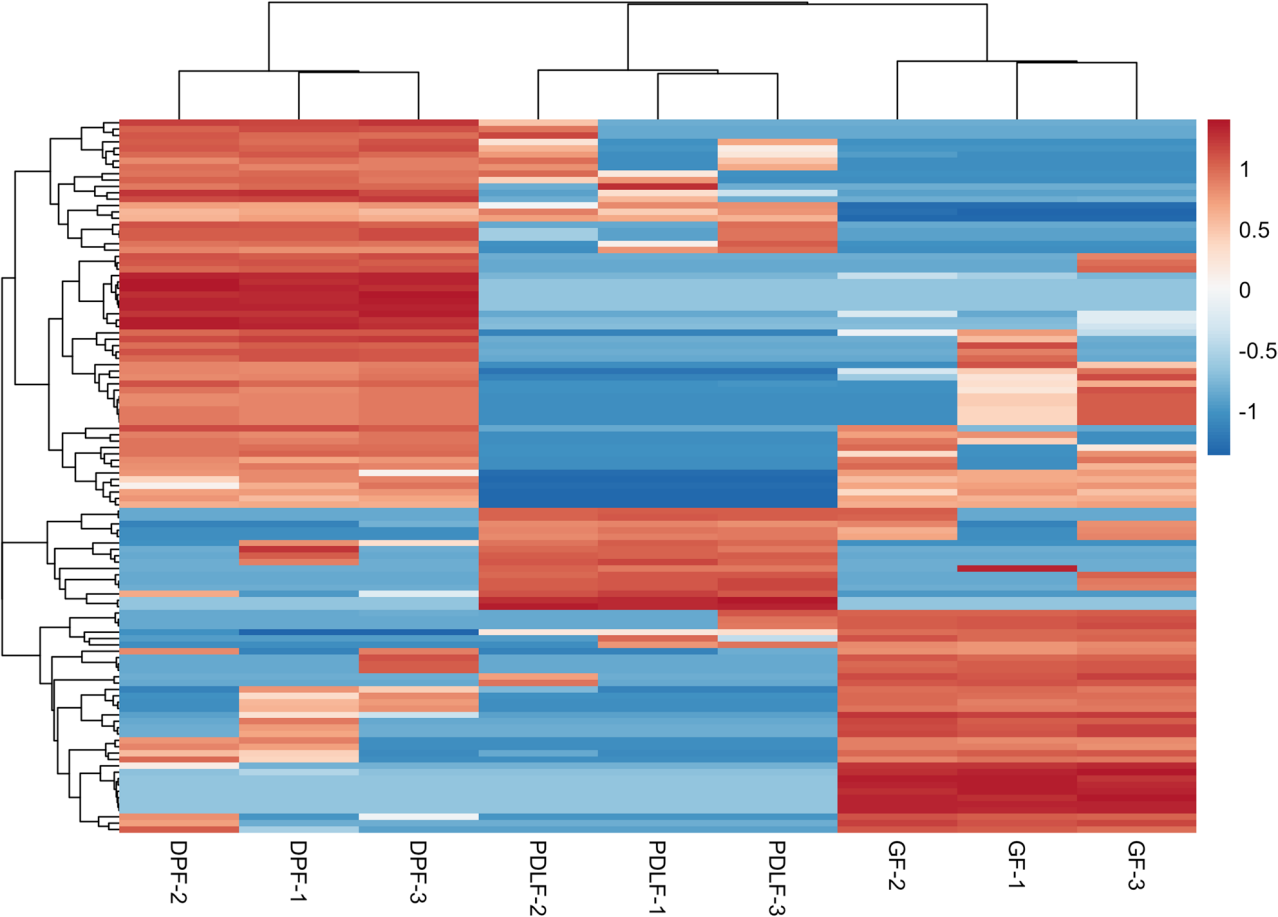
Heatmap Highlights Transcriptomic Signatures across Fibroblast-Derived Exosomes. Hierarchical clustering heatmap of exosomal RNA-seq data demonstrates distinct gene expression patterns across DPF, PDLF, and GF samples. TPM values were Z-score transformed and clustered based on gene-wise similarity. DPF samples formed a coherent cluster characterized by high expression of a large gene set, which was downregulated in GF samples. GF samples displayed an opposite expression pattern and formed a separate cluster, while PDLF samples showed intermediate expression, clustering between DPF and GF. These findings indicate the presence of distinct and gradated transcriptomic profiles among the three fibroblast-derived cell types

Figure 8 presents a hierarchical clustering heatmap based on RNA-seq expression profiles, revealing distinct transcriptomic signatures among DPF, PDLF, and GF. TPM values were Z-score normalized across genes. The heatmap shows that DPF samples clustered together and exhibited a consistent pattern of high expression (red) in a large set of genes that were simultaneously downregulated (blue) in GF samples. Conversely, GF samples formed a separate cluster with a reciprocal expression trend, highlighting their unique transcriptomic identity. PDLF samples displayed an intermediate expression profile and clustered between DPF and GF, sharing partial overlap with each. This gradient-like pattern of expression reflects both lineage- and condition-specific transcriptomic shifts among the three fibroblast-derived cell populations.

### Coordinated expression of exosomal miRNAs and their targets across fibroblast types

An extended analysis of Table 3 highlights multiple exosomal miRNA–mRNA relationships characterized by inverse expression patterns across the three fibroblast types, consistent with canonical miRNA-mediated suppression.

**Table 3:** Integrated analysis of differentially expressed miRNAs and their putative target mRNAs in exosomes

	miRNA-seq				RNA-seq						smallRNA-seq				
	Gene ID	Regulation	Log FC	pval	Gene Symbol	Gene ID	Regulation	Gene Type	Log FC	pval (Corr)	GO Accession	Ensembl ID	Regulation	Log FC	pval
GF vs. PDLF	hsa-miR-660-5p	up	7.68	3.47E-05	XKR7	ENSG00000260903	down	protein_coding	-2.24	0.04787515	hsa-mir-660	ENSG00000207970	up	3.25	1.71E-05
	hsa-miR-199a-5p	down	-5.44	0.04044385	COL19A1	ENSG00000082293	up	protein_coding	5.02	0.01652274	none				
GF vs. DPF	hsa-let-7i-5p	up	1.35	0.00143582	RWDD1	ENSG00000111832	down	protein_coding	-5.73	0.02492121	hsa-let-7i	ENSG00000199179	up	1.75	0.010202067
	hsa-let-7i-5p	up	1.35	0.00143582	ESPL1	ENSG00000135476	down	protein_coding	-5.15	0.00739531	hsa-let-7i	ENSG00000199179	up	1.75	0.010202067
	hsa-let-7i-5p	up	1.35	0.00143582	SLC5A6	ENSG00000138074	down	protein_coding	-4.44	0.02715941	hsa-let-7i	ENSG00000199179	up	1.75	0.010202067
	hsa-miR-320b	up	1.05	0.00810318	MTRNR2L6	ENSG00000270672	down	protein_coding	-6.71	0.0155828	hsa-mir-320b-2	ENSG00000221406	up	1.28	0.002371082
	hsa-miR-1307-3p	up	1.14	0.01666039	SNRPD1	ENSG00000167088	down	protein_coding	-4.83	0.04116506	hsa-mir-1307	ENSG00000283867	up	1.60	0.015848106
	hsa-miR-30b-3p	up	7.35	3.53E-04	SNRPD1	ENSG00000167088	down	protein_coding	-4.83	0.04116506	none				
	hsa-miR-493-5p	up	6.49	1.76E-05	HMGXB4	ENSG00000100281	down	protein_coding	-5.80	0.00651718	none				
	hsa-miR-26b-5p	down	-1.36	0.03805623	MB	ENSG00000198125	up	protein_coding	6.03	0.04431253	none				
	hsa-miR-26b-5p	down	-1.36	0.03805623	PCSK1	ENSG00000175426	up	protein_coding	5.96	0.02807161	none				
	hsa-miR-26b-5p	down	-1.36	0.03805623	HSPD1	ENSG00000144381	up	protein_coding	5.49	0.01610026	none				
	hsa-miR-26b-5p	down	-1.36	0.03805623	SDE2	ENSG00000143751	up	protein_coding	5.20	9.77E-04	none				
	hsa-miR-26b-5p	down	-1.36	0.03805623	PRSS16	ENSG00000112812	up	protein_coding	5.20	0.01619959	none				
	hsa-miR-26b-5p	down	-1.36	0.03805623	NOX3	ENSG00000074771	up	protein_coding	4.01	0.00651718	none				
	hsa-miR-26b-5p	down	-1.36	0.03805623	PLCB4	ENSG00000101333	up	protein_coding	3.65	0.01316521	none				
PDLF vs. DPF	hsa-miR-122-5p	down	-8.18	2.47E-06	XPO5	ENSG00000124571	up	protein_coding	4.36	0.04901887	hsa-mir-122	ENSG00000284440	down	-3.09	0.003299831

This table summarizes the integrated analysis of miRNA-seq, small RNA-seq, and mRNA-seq datasets from exosomes derived from DPF, PDLF, and GF fibroblasts. Differentially expressed miRNAs (DEmiRs) were identified based on FDR-adjusted *p* < 0.05 and |log₂ fold change| > 1. Corresponding small non-coding RNAs were matched by accession or Ensembl ID. Putative mRNA targets were selected based on inverse expression patterns and validated interactions in miRTarBase. *p*val (Corr) indicates a p-value corrected for multiple testing

In the GF vs. PDLF comparison, hsa-miR-660-5p and its precursor (hsa-mir-660) were significantly upregulated [23], while its experimentally validated target gene XKR7 was downregulated (miRTarBase ID: MIRT510537) [24]. Conversely, hsa-miR-199a-5p was downregulated [25], accompanied by upregulation of its predicted target COL19A1 (MIRT401907) [26, 27].

In the GF vs. DPF comparison, hsa-let-7i-5p and its precursor (hsa-let-7i) were upregulated [28], while predicted targets RWDD1 (MIRT616367) [29], ESPL1 (MIRT065692) [30], and SLC5A6 (MIRT501112) [31] were downregulated. Additional examples include hsa-miR-320b (upregulated) targeting MTRNR2L6 (downregulated) [32, 33], and hsa-miR-1307-3p (MIRT677278) / hsa-miR-30b-3p (MIRT648299) (both upregulated) [34, 35], targeting SNRPD1 (downregulated) [36, 37]. hsa-miR-493-5p was also elevated [38], with a corresponding decrease in HMGXB4 (MIRT352642) [39]. In contrast, hsa-miR-26b-5p was downregulated [40], while multiple predicted targets, MB (MIRT029607) [41], PCSK1 (MIRT029995) [42], HSPD1 (MIRT618502) [43], SDE2 (MIRT564206) [44], PRSS16 (MIRT030266) [45], NOX3 (MIRT029921) [46], and PLCB4 (MIRT028848) [47], were upregulated.

In the PDLF vs. DPF comparison, hsa-miR-122-5p and its precursor (hsa-mir-122) were significantly downregulated in PDLF, with upregulation of its validated target XPO5 (MIRT706980) [48].

These coordinated expression patterns underscore fibroblast subtype–specific regulatory axes and suggest their functional involvement in apoptosis regulation, extracellular matrix remodeling, pluripotency maintenance, and osteogenic differentiation.

## Discussion

This study provides a comprehensive transcriptomic comparison of fibroblast-rich cell populations derived from GF, PDLF, and DPF. By using matched samples from the same donors, inter-individual variability was minimized. PCA and hierarchical clustering analyses revealed group-dependent transcriptomic profiles with partial overlap among fibroblast types, reflecting both their tissue-specific identity and inherent biological variability.

Differential expression, k-means clustering, and GO enrichment analyses revealed functional specializations: GF showed features of immune responsiveness and tissue remodeling; PDLF was enriched in genes related to anti-inflammatory signaling and tissue homeostasis; and DPF displayed MSC-like molecular signatures, suggesting enhanced regenerative potential. Among these cell types, DPF-derived exosomes may represent the most promising candidates for regenerative applications. Previous studies have reported regenerative applications of exosomes and stem-cell–derived products from dental pulp, periodontal ligament, and gingival tissues [1–4]. However, these studies primarily characterized differentiation potential and therapeutic applications, whereas our work provides a transcriptome-wide comparative analysis of matched fibroblast-rich populations and their exosomal RNA cargo, thereby extending previous knowledge by highlighting subtype-specific molecular programs and putative regulatory axes.

Furthermore, the MSC-like transcriptomic features observed in DPF-derived exosomes should be interpreted in relation to previous studies profiling exosomes isolated from purified dental MSC populations. Prior analyses of dental pulp–derived MSCs, periodontal ligament–derived MSCs, and gingiva-derived MSCs have reported enrichment of stemness-related and osteogenesis-associated miRNAs within their exosomal cargo. Several of the molecular features enriched in DPF-derived exosomes parallel regulatory signatures previously described in MSC-derived exosomes, including stemness-related and osteogenesis-associated miRNA pathways [34–41, 43]. These similarities suggest that DPF-derived exosomes may retain molecular programs characteristic of MSC populations, despite being isolated from fibroblast-rich cultures. However, differences in donor origin, cell purification strategies, and vesicle isolation protocols across studies currently limit direct comparison. A more precise evaluation will require future studies that analyze fibroblast-derived and MSC-derived exosomes under harmonized experimental conditions.

The top upregulated genes in each type were associated with unique biological roles.

In GF, TRPM8 [49–51] and PAX6 [52] were linked to inflammatory sensitivity and neural differentiation, while CAV2 [53, 54] and SNX27 [55] suggested roles in structural homeostasis. In PDLF, genes such as CLIC1 [56, 57], DDX46 [58], and BACH1 [59–61] indicated responses to inflammation and oxidative stress. In DPF, genes including BMP7 [62], CECR2 [63], and FKBP8 [64] pointed to pluripotency and regenerative readiness.

Exosomal RNA profiling further revealed cell-type–specific miRNA–mRNA regulatory axes. In GF-derived exosomes, the upregulation of hsa-miR-660-5p and downregulation of XKR7 suggest involvement in apoptosis regulation [23, 24]. In PDLF-derived exosomes, the downregulation of miR-199a-5p accompanied by increased COL19A1 expression pointed to extracellular matrix-related remodeling [25–27]. In DPF-derived exosomes, hsa-miR-1307-3p and hsa-miR-30b-3p targeting SNRPD1 [34–37], hsa-miR-493-5p targeting HMGXB4 [38, 39], and hsa-miR-26b-5p targeting MB and HSPD1 reflect mechanisms tied to stemness and osteogenesis [40, 41, 43].

Collectively, the nominated axes converge on several biological themes relevant to regeneration, stemness and epigenetic control, RNA-processing and proliferative demand, mitochondrial proteostasis and metabolic shifts during osteogenic commitment, extracellular-matrix remodeling, and apoptosis regulation. These observations are consistent with the MSC-like molecular profile of DPF and the homeostatic roles of PDLF and GF, although they should be interpreted as hypothesis-generating.

These miRNA–mRNA axes were selected based on inverse expression patterns, consistent with the canonical mechanism of miRNA-mediated post-transcriptional repression, in which upregulated miRNAs suppress the expression of their target mRNAs [21, 65, 66].

Notably, the lack of correspondence between intracellular and exosomal expression patterns supports the existence of selective miRNA packaging mechanisms, potentially mediated by RNA-binding proteins as suggested in previous studies [11, 16, 67], rather than a passive reflection of cellular profiles. However, because intracellular miRNA profiling was not performed in this study, the possibility remains that these inverse patterns reflect cellular RNA content rather than selective export.

A rigorous test of preferential packaging will require matched intracellular and exosomal profiles and enrichment analyses at the miRNA family/seed and mRNA class levels, which were beyond the scope of this small (*n* = 3) dataset. Accordingly, the present results should be viewed as exploratory evidence suggesting, but not conclusively proving, the presence of active sorting processes.

Although several key miRNA–mRNA axes were supported by bioinformatic predictions and validated interactions in public databases such as miRTarBase, experimental validation was not performed. Consequently, the proposed axes should be regarded as putative in oral fibroblasts. Future work employing orthogonal assays, such as miRNA mimic or inhibitor perturbation combined with target knockdown, overexpression, and rescue experiments, will be essential to establish the mechanistic relevance of these predicted interactions.

Exosome transfer experiments, as well as gain- and loss-of-function studies in recipient cells, will further clarify whether DPF-derived exosomes can modulate regenerative signaling in a biologically meaningful manner.

To limit spurious inferences, we prioritized miRNA–mRNA pairs with experimental support in miRTarBase and required significant, donor-consistent inverse trends (FDR < 0.05) with a minimum abundance threshold; pairs lacking such evidence are designated as putative. Even with these filters, and without intracellular miRNA profiling or orthogonal perturbation (e.g., reporter assays, gain-/loss-of-function tests), context dependence cannot be excluded. We therefore present these axes as hypothesis-level findings rather than definitive mechanistic conclusions.

Before any translational application, a stepwise path is required: confirm biological activity in standardized in vitro potency assays; demonstrate proof-of-concept efficacy and biodistribution in appropriate preclinical models; and establish a manufacturing/quality framework consistent with MISEV 2023 and Good Manufacturing Practice (GMP) [68–71], together with a focused safety evaluation (immunogenicity/coagulation and potential pro-fibrotic or pro-tumorigenic risks). Until such data are available, DPF-derived exosomes should be regarded as investigational candidates.

This study has several limitations. The sample size was small (*n* = 3) and limited to young Japanese donors, which may introduce donor-specific bias and restrict generalizability. Although a widely adopted exosomal RNA isolation kit was used, consistent with prior RNA-centric studies [19, 20], standard exosome characterization assays (transmission electron microscopy, nanoparticle tracking analysis, and Western blot) were not performed, which prevented definitive confirmation of exosome purity and identity.

While the inclusion of these assays would provide definitive confirmation of vesicle identity, such analyses could not be performed within the scope of this study due to sample constraints. Accordingly, our findings should be interpreted as exploratory transcriptomic insights awaiting further orthogonal exosome characterization and functional validation in future work.

Intracellular miRNA profiling and functional validation (e.g., tri-lineage differentiation, CFU assays, or direct comparisons of DPF- versus GF/PDLF-derived exosomes) were also lacking; thus, our conclusions remain at the omics level and require further validation. Partial overlap observed in the PCA plots may reflect donor variability as well as the heterogeneous composition of fibroblast-rich populations. Finally, we intentionally analyzed fibroblast-rich cultures containing a minor MSC fraction to capture clinically relevant transcriptomic signatures, but the MSC proportion was not quantified.

Taken together, these limitations underscore that the present findings should be regarded as preliminary and exploratory. Future studies should aim to validate the identified transcriptomic and exosomal signatures in larger and more diverse donor cohorts, perform comprehensive exosome characterization following MISEV 2023 standards, and conduct orthogonal functional assays.

Given the absence of functional validation and the reliance on omics-based inference, the present work should be regarded as a preliminary study intended to generate testable hypotheses for future mechanistic analyses.

In conclusion, fibroblast types from oral tissues exhibit distinct transcriptomic and exosomal profiles that reflect differences in differentiation, immune responses, and regenerative potential. Among them, DPFs displayed pronounced MSC-like molecular signatures, suggesting their potential as a source of therapeutic exosomes. These findings support the feasibility of developing fibroblast-derived, exosome-based regenerative strategies without requiring pure MSC isolation.

## Data Availability

All datasets supporting the findings of this study are publicly available in Figshare under the DOI: 10.6084/m9.figshare.29803016. The repository includes raw read count matrices, quantified and normalized datasets from exosomal RNA-seq, miRNA-seq, and small RNA-seq, as well as differential expression results and filtered gene lists.

## Abbreviations

BP: Biological process
CC: Cellular component
DEG: Differentially expressed gene
DPF: Dental pulp fibroblast
EVs: Extracellular vesicles
FBS: Fetal bovine serum
FDR: False discovery rate
FC: Fold change
GF: Gingival fibroblast
GMP: Good manufacturing practice
GO: Gene Ontology
LRT: Likelihood ratio test
MF: Molecular function
miRNA: MicroRNA
MISEV: Minimal information for studies of extracellular vesicles
MSC: Mesenchymal stem cell
PCA: Principal component analysis
PDLF: Periodontal ligament fibroblast
RNA-seq: RNA sequencing
TMM: Trimmed mean of M-values
TPM: Transcripts per million
